# Elkoplasty, or Anaplasty Applied to the Treatment of Old Ulcers

**Published:** 1854-12

**Authors:** 


### ECLECTIC DEPARTMENT,

### AND SPIRIT OF THE MEDICAL PERIODICAL PRESS.

### *Elkoplasty, or Anaplasty applied to the Treatment of Old Ulcers.*

By Frank H. Hamilton, M. D.

Some writer has said, "old ulcers in 1830 will be old ulcers in 1860," which, not to be understood always in a literal sense, was intended only to express, in a brief and pertinent form, the proverbial obstinacy of this class of sores.

In most cases, the integument has been broken and destroyed by ulceration, and then, usually, bad health, or, perhaps, enlarged veins, have helped to perpetuate the lesion. In other cases, however, the ulcers are directly in consequence of some severe lacerating injuries, which have at once torn away the skin beyond the power of nature to repair; and that although the health of the body and of the limb may be perfect. In such cases, the refusal of the ulcers to heal is entirely owing to the extensive loss of integument.

Actual loss of skin is repaired by one or both of two processes. By the development of new, from or upon the free margin of the old skin, or by the contraction of the granulations and of the cicatrix, in consequence of which, the adjacent skin is drawn towards the chasm, and made, as it were, to slide over and cover it in.

This rule admits of but few exceptions. Occasionally, after a very long delay, the granulations acquire the power of forming new skin at various and isolated points of the sore. This process may now and then be observed in the healing of extensive burns, or, perhaps, in the closing up of an ulcer whose surface is excluded from the air. New skin may even find a matrix in the periosteum, as I have witnessed, and maintained several years since. (*Buffalo Medical Journal*, vol. vii. p. 205.) But the conditions are very rare under which these exceptions can occur. The rule remains as we have stated, and if ulcers are not closed by either the projection of new skin from the margins of the old, or by the contraction of the granulations and cicatrix, then, usually, they must ramain open. To the action of both of these processes there is, however, a limit. The formative power of the old skin does not extend beyond a few lines. The new vessels, becoming more and more attenuated as they stretch inward from the periphery, lose, at length, the power of generating epithelial cells, or, if formed, they are too imperfectly organized to sustain an existence, and they crumble away from the slightest provocation. Slowly, but perceptibly, the opaque diaphragm proceeds to shut in the granulations, and for a long time encourages a hope that a cure is to be accomplished. But just when the work is almost consummated, a rapid disintegration sweeps away in a few hours the patient labor of many months. Again and again the reluctant labor is renewed, and as often suddenly, and without provocation, is it arrested and broken up. At the same time the granulations have ceased to condense, and the cicatrix to contract, either because these actions have attained their natural limits, or because the adjacent skin has reached its utmost tension, and affords effectual resistance to further stretching. Here the process of closure forever ends, and the "old ulcers of 1830 will be old ulcers in 1860."

Nature has done its utmost, and hitherto art has failed to complete the work.

I beg to suggest a procedure, which, hereafter, in some unfortunate cases of this class may deserve a trial.

I propose to close the ulcer by an operation of anaplasty. In short, to imitate one of the processes of nature, by sliding in old skin to repair a waste, where the process of forming new skin has ceased, and been finally given up.

If we seek to obtain this supply from the neighborhood of the ulcer, around which the skin has already reached its utmost tension, we shall only substitute one ulcer for another. We must, therefore, generally look to the opposite limb, or to the limb of some other person, for the material with which the transplantation or engrafting is to be made.

The mode of accomplishing this, will not differ materially from that which has been generally adopted in anaplasty from remote parts, except that the ulcerated surface ought to be exercised freely before the new skin is laid upon it.

By this means, I hope, gentlemen, not only to supply an amount of skin equal to the size of the piece transferred, but to furnish, also, a nucleus from which additional skin shall be formed. I hope to establish a new center of life—an oasis—from whose outer verge a true and healthy vegetation shall advance in every direction over the exhausted soil.

It is not improbable, also, that the graft will itself expand, or be drawn centrifugally by the contraction of the surrounding granulations and cicatrix, conversely, as the skin about the ulcer had before been stretched and drawn centripetally, by a similar action of the granulations and cicatrix situated within its free margin, so that, after a time, it will cover more space, independent of any actual growth, than it did originally. The opposite of this happens usually in anaplasty, and would occur here, did the flap equal or exceed in size the wants of the parts to be supplied. The flap would contract, thicken, and project itself above the surface. But in old ulcers, it will generally be found impossible to furnish a direct supply of integument equal to the loss. A deficiency must probably still exist, and sufficient, it is believed, to determine in the transplanted skin a necessity of expansion.

The value and practicability of these views are, I trust, in a measure established by the results, in the case which I shall now take the liberty of bringing before you.

You will excuse me, however, if I detain you a moment longer to explain to you that, so long as eight years since, I proposed the same operation, and had anticipated most of the results which I have now actually obtained.

In the report of my surgical clinic, for 1846, at Geneva Medical College, published in the *Buffalo Medical Journal*, vol. ii. p. 508, occurs the following passage:—

"*Indolent ulcer.* M—— of Geneva.—This lad, now about fifteen years old, had the right leg and part of the thigh terribly lacerated, and almost deprived of its integument, by a threshing-machine, eight years ago. The wound has never closed entirely, but an indolent ulcer of great extent exists, surrounded by a broad margin of hard integument, from which sometimes new skin will form, and then it will rapidly crumble away, and the ulceration will extend, perhaps, beyond its original bounds. Thus it has continued to partially close and again open, during all this time; meanwhile, the health and strength of the lad have remained excellent, but the leg has become bent at the knee, and he walks with a halt. Two years ago Dr. Hamilton took a cast of the ulcer, which is now seen to correspond almost precisely with its present extent.

"Dr. Hamilton and others having tried almost every plan of treatment which would offer a prospect of success, and having so completely failed, as Dr. Hamilton believes, because the indurated margin, near two inches in breadth—all around the sore, is incapable of projecting from itself sound skin, the Dr. has proposed to the boy a plastic operation, with the view of planting upon the center of the ulcer a piece of new and perfectly healthy skin. He proposes to take this from the calf of the other leg (having secured the two together), not intending to cover the whole sore, but perhaps two or three square inches, which he believes will be enough to secure the closure of the whole wound in a short time."

Two years before the date of this clinic, when I took the cast alluded to in the above report, I had made the same proposition to the lad, and when he declined submitting to it, I appealed to his father, who was a worthless inebriate, to allow me to secure one of his legs to his son's, that I might make the transplantation from him. In no other way, I assured him, could he so much benefit his family.

I need scarcely say that permission was never obtained, and that I have never found an opportunity of determining the practicability of my suggestions until during the last year, and in the person of the man who is now before you.

The following is the report of the case, copied, in part, from the Hospital Records :

Horace Driscoll, aged 40 years; Irish laborer; had the skin and flesh extensively torn from the right leg by a dirt car, on the 3d of November, 1852. He has been in the hospital most of the time since then until now. The wound has nearly healed several times, but never entirely ; after exercise the whole would give way, and the ulcer again extend itself completely around the leg.

*Jan.* 21, 1854. I made the following operation:

The patient was laid upon his belly, upon the operating table before the class. A flap of skin measuring seven inches by four was then raised carefully from the calf of the opposite leg, extending in depth through the cutaneous and celluloadipose textures, until the fascia was in sight. Its remaining attachment to the body was by a broad and thick base. The hæmorrhage was slight ; no vessels were tied. Lint, spread on both surfaces with simple cerate, was laid between the flap and the surface from which it had been detached, other pledgets of lint similarly covered were placed on the outer surface, while over all and around the entire limb was wrapt a large mass of cotton batting, secured in place by a lightly turned roller.

He was then laid in bed and perfect quietude enjoined.

*Jan.* 22d.—During the night the wound has bled until the patient looks pale from the loss. The bleeding has now ceased.

*Feb.* 4th.—Two weeks since the flap was raised. The patient has had to be sustained with beer, his appetite having failed very much since the operation. The flap has been dressed in the same manner as at first, nearly every day. It looks healthy. No part of it has sloughed.

To-day the operation was recommenced before the class, by dissecting out the granulations and part of the cicatrix from the diseased leg, and thus forming a deep bed of the size and shape of the flap as it now appeared, both contracted and thickened. The flap was then made raw again on its margins, and its lower surface was shaved off, with the double purpose of removing the granulations, and of diminishing its excessive thickness. When the bleeding had ceased, the left leg was carried across the right, so that the tendo-Achilles and heel of the left leg rested upon the instep and ankle of the right—a thick cotton pad being interposed to prevent painful pressure. The flap was now brought snugly into its new bed, on the right leg, and well secured with interrupted sutures, a moderate compress, and roller. The two limbs were further secured immovably to each other by bands, and protected at various points by well made compresses, and the wounds carefully covered with lint spread with cerate.

*Feb.* 5th.—The wound has bled again, as after the first operation, although ice was applied diligently from the moment the dressings were completed. Much pressure was regarded as inadmissible. Bleeding ceased when he became faint, about three hours after the operation.

*Feb.* 18th.—Two weeks since the last operation, and four weeks from the first. Patient has required to be sustained constantly with beer and nourishing diet. His appetite still remains bad. Bowels have not been moved in two weeks. He has not suffered much pain, only fatigue. To-day the base was separated from the left leg, the flap having united through most of its edges and under surface, to the opposite leg. No bleeding of consequence followed. The parts were thoroughly washed and dressed with ung. basil. and a snug roller applied. Ordered sulph. mag. ℥j.

*Feb.* 19th.—No movement of bowels. Repeat sulph. mag.

*Feb.* 20th.—One corner of the extreme end of the flap is beginning to slough.

*Feb.* 21st.—Bowels have moved. Sloughing of flap continues. Ordered yeast poultice.

*Feb.* 25th.—Line of demarcation formed, insulating about one inch and a half of the flap, at the corner where the sloughing commenced.

Beyond this the sloughing never extended. The surfaces continued to close, and about one hundred days after the flap was laid down the healing was finally consummated, and now after a lapse of nearly three months, during which he has been acting as a subordinate dresser at the hospital, the ulcer has not re-opened or shown any tendency that way.

The wound made by the removal of skin from the left leg was completely healed over in about the same length of time as the ulcer on the right, and the whole left limb is now as sound and as perfect as before the operation.

Driscoll is, however, at present, by no means a well man. His health has suffered considerably from his long illness, and from his prolonged confinement in bed, which dates from the time of the accident, through most of the period, up to the time of the closing of the wounds since the operation. The cicatrix around the new skin is tender, and especially at one point where several pieces of bone exfoliated soon after the accident, and precisely over which, unfortunately, the sloughing of the flap took place. The ankle is also somewhat stiffened by the contraction of the skin, and of the gastrocnemii and tendo-Achilles, which latter were seriously involved in the original injury. These, however, are conditions which the operation did not propose to remedy, at least only in a small degree, or they are temporary accidents, and will certainly yield to time and careful use. If they were to continue, however, it will not be denied that, in the permanent sealing up of a sore, which, but for this operation, must probably have remained open during life, he is amply repaid for all that he has suffered at my hands. I venture to predict that, within one year from this time, he will be able to labor nearly or quite as well as before the accident.

On the 12th of March, five weeks after the flap had been transplanted, it had united by adhesion to the adjacent skin, through about one half of its circumference. The other half was surrounded by a border of granulations and of new skin, varying in breadth from one to ten or fifteen lines; but only at a few points was the bridge of new skin complete. It was especially noticed that nearly all, probably nine-tenths, of this new skin had sprung from the margins of the flap, and only the remaining fraction from the adjacent cicatrix; demonstrating that after transplantation and complete separation from the parent limb, its vitality was unimpaired, and that its re-productive power, if I may so speak, was vastly superior to the re-productive power of the old cicatrix.

You may notice to-day, also, that since the cicatrization was completed, the cicatrix formed by growth from the flap, has contracted; and, that, in consequence of this contraction, the flap has become expanded, or been stretched outward, and its surface has become flattened and firm, whereas, it was, at first, and for a long time, elevated above the surrounding skin, and flabby

*Summary:*—

1st.—Ulcers, accompanied with extensive loss of integument, do generally refuse to heal, whatever may be the health of the body or of the limb.

2d.—Anaplasty will sometimes succeed in accomplishing a permanent cure, and especially where the health of the body and of the limb are perfect, and where, by inference, the refusal to heal is alone attributable to the extent of the tegumentary loss.

3d.—The graft must be brought from a part quite remote; generally from an opposite limb, or from another person.

4th.—If smaller than the chasm which it is intended to fill, the graft will grow, or project from itself new skin to supply the deficiency.

5th.—It is not improbable that the graft will expand during the process of cicatrization at its margins, but especially for a time after the cicatrization is consummated.

6th.—In consequence of one or of both of these two latter circumstances, it will not be necessary to make the graft so large as the deficiency it is intended to supply.—*From N. Y. Journal of Medicine and Collateral Sciences.*

